# Phytochemical Composition and Cytotoxic Effects on Liver Hepatocellular Carcinoma Cells of Different Berries Following a Simulated In Vitro Gastrointestinal Digestion

**DOI:** 10.3390/molecules23081918

**Published:** 2018-08-01

**Authors:** Francesca Giampieri, Sadia Afrin, Derek Stewart, Gordon J. McDougall, Rex Brennan, Lesley Blyth, Massimiliano Gasparrini, Luca Mazzoni, Franco Capocasa, Josè Miguel Alvarez-Suarez, Stefano Bompadre, Pedro Nogueira Brás de Oliveira, Claudia N. Santos, Manuel Masias, Pablo Agudo, Jorge Crespo, Bruno Mezzetti, Tamara Y. Forbes-Hernández, Maurizio Battino

**Affiliations:** 1 Dipartimento di Scienze Cliniche Specialistiche ed Odontostomatologiche-Sez. Biochimica, Facoltà di Medicina, Università Politecnica delle Marche, 60131 Ancona, Italy; f.giampieri@univpm.it (F.G.); dolla.bihs@gmail.com (S.A.); m.gasparrini@univpm.it (M.G.); 2 The James Hutton Institute, Dundee DD2 5DA, UK; Derek.Stewart@hutton.ac.uk (D.S.); Gordon.McDougall@hutton.ac.uk (G.J.M.); rex.brennan@hutton.ac.uk (R.B.); lesley.blyth@hutton.ac.uk (L.B.); 3 Department of Engineering and Physical Sciences, Heriot-Watt University, Edinburgh EH14 4AS, UK; 4 Division of Food Production and Society, Norwegian Institute of Bioeconomy Research, Pb 115, NO-1431 Ås, Norway; 5 Dipartimento di Scienze Agrarie, Alimentari e Ambientali, Università Politecnica delle Marche, Via Ranieri 65, 60131 Ancona, Italy; l.mazzoni@univpm.it (L.M.); f.capocasa@univpm.it (F.C.); b.mezzetti@univpm.it (B.M.); 6 Facultad de Ingeniería y Ciencias Agropecuarias (FICA), Grupo de Investigación en Biotecnología Aplicada a Biomedicina (BIOMED), Universidad de Las Américas, José Queri y Ave. de los Granados, Quito 170513, Ecuador; jose.alvarez@udla.edu.ec; 7 Dipartimento Scienze Biomediche e Sanita’ Pubblica, Facoltà di Medicina, Università Politecnica delle Marche, 60131 Ancona, Italy; s.bompadre@univpm.it; 8 Instituto Nacional de Investigacao Agraria e Veterinaria (INIAV), 2780-113 Carcavelos, Portugal; pedro.oliveira@iniav.pt; 9 iBET, Instituto de Biologia Experimental e Tecnológica, Apartado 12, 2781-901 Oeiras, Portugal; dsb.lab.itqb@gmail.com; 10 Center for Nutrition & Health. CITICAN, Universidad Europea del Atlántico, Parque Científico y Tecnológico de Cantabria C/Isabel Torres 21, 39011 Santander, Spain; Manuel.masias@uneatlantico.es (M.M.); pablo.agudo@uneatlantico.es (P.A.); jorge.crespo@uneatlantico.es (J.C.)

**Keywords:** berry, polyphenols, in vitro gastrointestinal digestion, bioavailability, cytotoxicity

## Abstract

Berry fruits are rich in nutrients and polyphenols, providing potential health benefits. Understanding the factors that affect their bioavailability is becoming of utmost importance for evaluating their biological significance and efficacy as functional food. In this study, the phytochemical composition and the total antioxidant capacity of different varieties of five berries (blackberry, blackcurrant, blueberry, raspberry, and strawberry) were evaluated after an in vitro gastrointestinal digestion process. The cultivar of each berry that showed the higher content of total phenols and flavonoids was selected to study its cytotoxic effect on human hepatoma cells. Digestion resulted in a high reduction (*p* ˂ 0.05) of total phenolic, flavonoid and anthocyanin contents and total antioxidant capacity, in the “IN” samples compared to the “OUT” extracts, which represent the “serum-available” and the “colon-available” fractions, respectively. Incubation of the digested fraction for 24 h didn’t exert any effect on cellular viability, while a dose- and time-dependent cytotoxicity was observed after 48 h and 72 h of incubation for all the berries analyzed. Our results suggest that the approach proposed in this work may represent a rapid tool for evaluating and identifying new berries with increased phytochemical bioavailability, highlighting their antiproliferative agents after an in vitro digestion.

## 1. Introduction

The importance of diet on human health has been widely recognized worldwide. It is commonly accepted that the regular consumption of plant-based food is strongly correlated with a high intake of bioactive compounds that play a crucial role in the prevention of a wide range of diseases, such as cancer, cardiovascular disorders, and other chronic degenerative pathologies, as well as with the general health benefits they provide [1,2,3]. Berries may be a relevant component of these healthy diets, since they are fruits rich in nutritive compounds, including minerals, vitamins, dietary fibers, and in non-nutritive elements, especially polyphenolic phytochemicals [4,5]. The most common berries include blackberry (*Rubus* sp.), blackcurrant (*Ribes nigrum*), blueberry (*Vaccinium corymbosum*), raspberry (*Rubus idaeus*), and strawberry (*Fragaria x ananassa*), which are usually consumed in fresh and processed forms in the human diet all around the world. The principal group of phytochemicals in berry are polyphenols, comprising flavonoids (anthocyanins, flavonols, flavones, flavanols, flavanones, and isoflavonoids), stilbenes, tannins, and phenolic acids, which are reported to strongly contribute to the health effects of berry consumption against human diseases [6,7,8]. In recent years, it has become of utmost importance to improve the knowledge on polyphenol bioavailability, in order to understand the possible mechanisms of their health effects and to deeply characterize the impact of berries in human healthy life [9,10].

Bioavailability is a complex process which depends on many factors, including the release of nutrients/phytochemicals from the food matrix, their digestive stability, and their efficiency of transepithelial passage [11]. Because of glucuronidation/sulphation of free hydroxyl groups present in the chemical structures of the different compounds via the xenobiotic metabolism pathways, the bioavailability of polyphenols is rather low, not exceeding the plasma concentrations of 10 μM, and varies significantly from one compound to another [12,13]. However, after a meal rich in vegetables and fruits, dietary polyphenols can reach the gastrointestinal (GI) tract at great concentrations, where they exert an important role in protecting from oxidative stress and in retarding the onset of different types of cancer [14]. In vitro digestive models are currently used to predict, even if in a simplified manner, the polyphenol behavior in simulated digestive processes of the GI tract, providing significant information on the stability and putative modifications of polyphenols under GI conditions [15,16].

It is well known that berry polyphenol composition is highly influenced by the genotype: each type of berry and each type of cultivar have different profiles and contents of bioactive compounds [17,18,19,20]. These features define the nutritional quality for the fresh market and the stability of the phytochemical composition after long shelf-life or different processing treatments [21]. However, up to now very few studies are available on the stability of polyphenols after human digestion, and, since evidence of the potential health-promoting and disease-preventing effects of the berry phytochemicals continues to accumulate, it is becoming crucially important to understand the nature of their bioavailability, absorption and metabolism. 

The aim of the present study was to assess the bioacessability of different cultivars of five berries (blackberry, blackcurrant, blueberry, raspberry, and strawberry) using an in vitro digestion procedure that mimics the physiochemical and biochemical conditions encountered in the GI tract. In particular, we evaluated and compared the phytochemical composition (total phenolic, flavonoid and anthocyanin contents) and the total antioxidant capacity (through the Trolox Equivalent Antioxidant Capacity (TEAC) and Ferric Reducing Antioxidant Power (FRAP) assays) of the different digested samples, taking into account the “IN” and the “OUT” fractions, which represent the “serum-available” and the “colon-available” fractions, respectively. In addition, the cultivar of each berry that showed the higher content of total phenolic compounds and flavonoids was selected to test their cytotoxic effects on human hepatoma cells (HepG2), the most used cellular model for in vitro cytotoxicity studies given the simplicity of its use, culture and relatively low cost compared to primary human hepatocytes.

## 2. Results and Discussion

### 2.1. Berry Phytochemical Contents after In Vitro Digestion

In the present work, the phytochemical composition of the different berries was analyzed after the in vitro digestion process. Table 1 shows the TPC, Flavo and ACY of the different fractions, where IN samples represent the bioavailable fraction, that is the part that potentially reaches the blood circulation from the intestine, while OUT samples represent the fraction that is least bioavailable, which is eliminated through feces or metabolized by colon microbiota. 

Significant differences (*p* ˂ 0.05) were found between the diverse berries and between the “IN” and the “OUT” fractions of each berry. In particular, the OUT samples always presented the highest contents of TPC, Flavo, and ACY than the IN samples, in most cases with double values (*p* ˂ 0.05). 

Regarding the “IN” fractions, blackberry and black currant possessed the highest (*p* ˂ 0.05) total phenolic contents (TPC), followed by strawberry and raspberry; the lowest concentration (*p* ˂ 0.05) was registered for blueberry. Black currant also presented the highest (*p* ˂ 0.05) flavonoid content (Flavo), followed by blueberry and blackberry; the lowest contents (*p* ˂ 0.05) were found for raspberry and strawberry. Again, black currant had the highest (*p* ˂ 0.05) contents of anthocyanins (ACY), followed by blackberry, raspberry, strawberry, and blueberry. Black currant showed the highest value of total antioxidant capacity (TAC) (*p* ˂ 0.05), measured by TEAC assay, followed by raspberry (*p* ˂ 0.05), and by blackberry and strawberry, which showed similar values; the lowest value was found for blueberry (*p* ˂ 0.05). With FRAP method, black currant possessed the highest TAC (*p* ˂ 0.05), followed by strawberry and blackberry; blueberry and raspberry presented the lowest ones (*p* ˂ 0.05). In summary, black currant was the berry with the highest contents of polyphenols and the highest TAC for IN fractions. 

Concerning the “OUT” samples, blackberry possessed the highest content (*p* ˂ 0.05) of TPC, followed by black currant and strawberry (*p* ˂ 0.05); the lowest contents (*p* ˂ 0.05) were registered for blueberry and raspberry. Blueberry presented the highest (*p* ˂ 0.05) content of Flavo, followed by blackberry, black currant, and strawberry; the lowest content (*p* ˂ 0.05) was found for raspberry. As the “IN” fractions, black currant had the highest (*p* ˂ 0.05) content of ACY, followed by blackberry, raspberry and strawberry; the lowest content was found for blueberry (*p* ˂ 0.05). Black currant showed the highest TAC (*p* ˂ 0.05) measured by TEAC assay, followed by blackberry and raspberry, which presented similar values; blueberry had the lowest (*p* ˂ 0.05) values. With the FRAP method, the highest values were found for strawberry and black currant, followed by blackberry (*p* ˂ 0.05), blueberry (*p* ˂ 0.05) and raspberry which showed the lowest (*p* ˂ 0.05) concentration.

Significant differences (*p* ˂ 0.05) were also found between the diverse cultivars of each berry (Table 1). In particular, “Loch Ness” was the blackberry cultivar with the highest contents of total phenol, flavonoid, and anthocyanin (*p* ˂ 0.05) in both the “IN” and “OUT” fractions. Among black currant, “Ben Finlay” presented the highest content of total phenolic and flavonoid compounds (*p* ˂ 0.05) in the “IN” and “OUT” samples, while the highest (*p* ˂ 0.05) content of ACY were found for “Ben Starav” “IN” and “OUT” extracts. “Misty” cultivar was the blueberry with the highest (*p* ˂ 0.05) content of TPC and Flavo (*p* ˂ 0.05) in both the “IN” and “OUT” fractions, while “Georgiagem” presented the highest content of ACY. For raspberry, “Glen Ericht” was the cultivar with the highest content of TPC and Flavo in both “IN” and “OUT” fractions, followed by “Tulameen” variety. Finally, among strawberry, “Romina” presented the highest content of TPC and Flavo, while AN00.239.55 the highest content of Flavo. Interestingly, “Bianca” showed the highest (*p* ˂ 0.05) content of TPC and Flavo, together with “Romina” and “AN00.239.55,” respectively.

In addition, significant differences were outlined also between the IN and OUT fractions of each variety (Table 1). In particular, the OUT samples always presented higher contents in TPC, Flavo and ACY than the IN samples, in most cases with double values (*p* ˂ 0.05).

### 2.2. Berry Total Antioxidant Capacity after In Vitro Digestion

The total antioxidant capacity of the different cultivars of each berry was evaluated after the in vitro gastrointestinal digestion process by the TEAC and FRAP assays (Table 2).

As for the phytochemical characterization, significant differences (*p* ˂ 0.05) were outlined among the different varieties of each berry and between the “IN” and “OUT” fractions of each variety. In particular, for raspberry “Loch Ness” showed the highest TAC (*p* ˂ 0.05) measured by TEAC and FRAP assays in the IN fraction, while R. brigantinus and R. vagabundus presented the highest (*p* ˂ 0.05) values in the “OUT” fraction. “Ben Finlay” and “Ben Starav” showed the highest TAC (*p* ˂ 0.05) measured with the two methods both in the “IN” and “OUT” samples for black currant; “Misty” and “Glen Ericht” were the variety with the highest (*p* ˂ 0.05) TAC, both in the “IN” and “OUT” fraction, for blueberry and raspberry respectively. 

Surprisingly, *F. vesca*—white was the strawberry cultivar with the highest values (*p* ˂ 0.05) of TAC measured with the two methods both in the “IN” and “OUT” extracts; these high values can be due to the high levels of ellagitannins present in this variety. In addition, the “OUT” samples always presented higher concentrations than the IN samples, confirming the strong correlation between the phenolic content and the total antioxidant capacity of food matrices.

### 2.3. Berry Cytotoxicity after In Vitro Digestion

**Figure 1:**
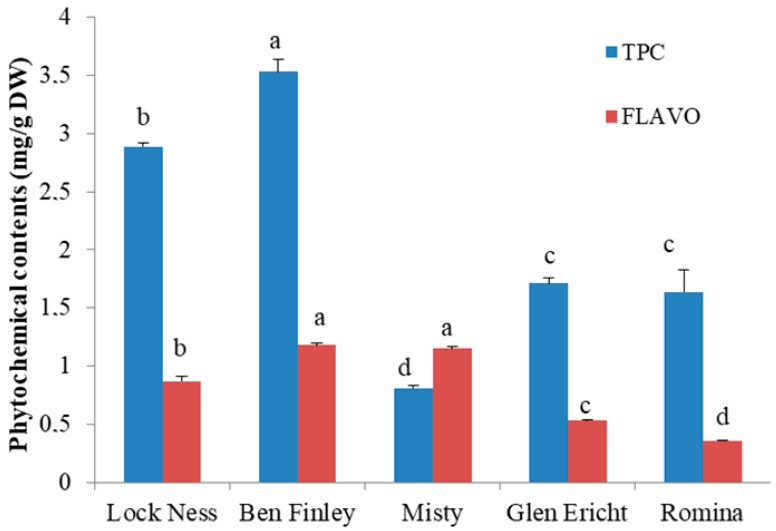
Phytochemical composition of the berry “IN” fractions selected for the in vitro study. Data are expressed as mean ± SD. Columns labeled with different letters are significantly different (*p* < 0.05). Abbreviations: TPC, total phenolic content; Flavo, total flavonoid content; DW, digested weight.

On the basis of results obtained for the phytochemical composition, the “IN” fraction of a cultivar of each berry was selected for the in vitro study: “Loch Ness” for blackberry, “Ben Finlay” for blackcurrant, “Misty” for blueberry, “Glen Ericht” for raspberry, and “Romina” for strawberry (Figure 1).

**Figure 2:**
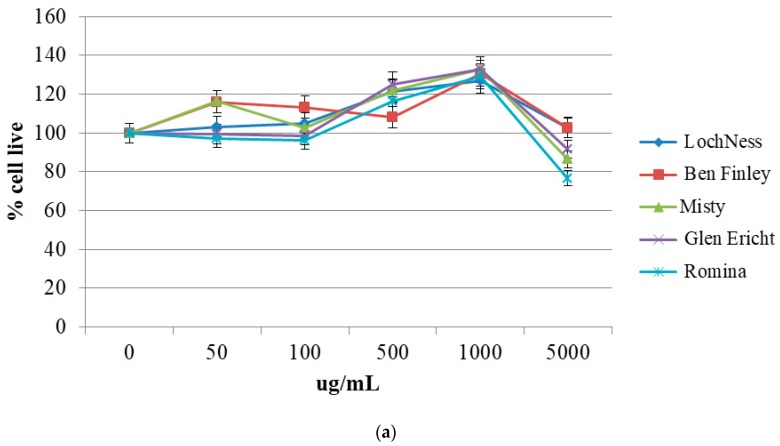

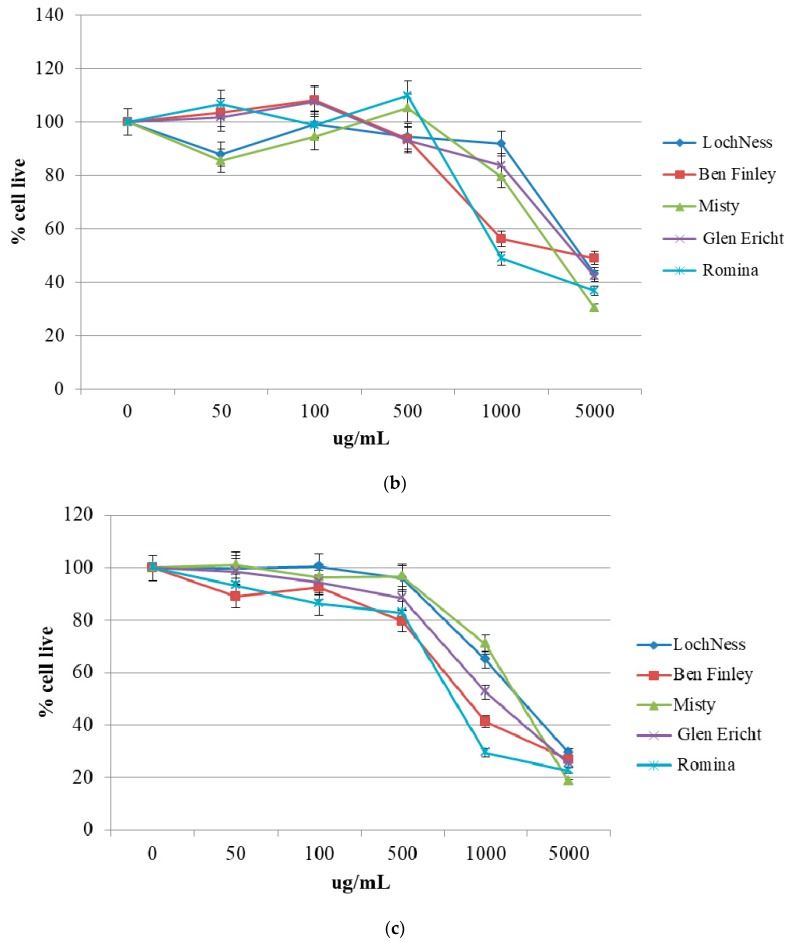
Viability of HepG2 determined by 3-(4,5-dimethylthiazol-2-yl)-2,5-diphenyltetrazolium bromide (MTT) assay. Cells were treated with various concentrations of berry digested extracts (0–5000 µg/mL) for 24 h (**a**), 48 h (**b**) and 72 h (**c**). Data are expressed as mean ± SD for eight replicas (*n* = 8) of three independent experiments.

The “IN” samples were evaluated for their antiproliferative activity on HepG2 cells. Cell viability was analyzed after 24 h, 48 h, and 72 h of exposure to media containing increasing levels of digested berry extracts (0–5000 µg DW/mL) using the MTT (3-(4,5-dimethylthiazol-2-yl)-2,5-diphenyltetrazolium bromide) assay (Figure 2).

Results showed a dose- and time-dependent cytotoxic effect of the “IN” fractions: after 24 h of incubation, they did not exert any effect on cellular viability at any concentrations, while after 48 h of incubation, cell vitality began to decrease at the concentration of 500 µg/mL especially for blackcurrant, blackberry and raspberry. At 72 h, cell viability decreased for all the berries analyzed, especially at higher concentrations. In particular, after 48 h of incubation, the most cytotoxic effects were registered for black currant (“Ben Finlay”) and strawberry (“Romina”), which led cell vitality near to 50% at 1000 µg/mL, followed by blueberry (“Misty”) and raspberry (“Glen Ericht”), which led the viability closer to 80%; the less cytotoxic berry was blackberry (“Loch Ness”), with a viability of about 90% at 1000 µg/mL. As expected, a similar trend was found after 72 h of incubation: strawberry (“Romina”) and black currant (“Ben Finlay”) were again the most cytotoxic berries (29% and 41% of viability at 1000 µg/mL, respectively), followed by raspberry (52% of viability at 1000 µg/mL); blackberry and blueberry exerted the weaker effect berries (65% and 71% of viability at 1000 µg/mL, respectively). 

## 3. Discussion

Berries are well known to possess a wide range of phytochemicals such as flavones, flavonols, anthocyanins, and phenolic acids [6,7,8], which ameliorate cellular oxidative stress and prevent the onset and development of chronic degenerative diseases [4,5,22]. It is clear that foodstuffs, crossing the GI tract, are exposed to different conditions before providing specific health benefits. Consequently, some functional nutrients and phytochemicals are transformed into other compounds with varied bioactivity [23]. Although some studies have evaluated the composition and the functional activities of some berries after the in vitro GI digestion process, it is necessary to deepen our knowledge on their bioacessability in order to underline the real effect of berry consumption on human health [24,25,26,27,28,29,30]. In the last few years, in vitro digestion methods for simulating the GI conditions have been broadly employed, since they are faster and cheaper than the in vivo analyses on animal or humans [16,31]. Even if the results obtained in animal or human studies usually may provide the most accurate results, the evaluations of bioacessability using the in vitro digestion model are strongly correlated with the results from animal or human studies, as previously reported [32]. For these reasons, in the present work, the phytochemical composition of the different berries was analyzed after the in vitro digestion process (Table 1). We found that the OUT samples, representing the “colon-available” fractions, showed higher values of TPC, Flavo and ACY, compared to the IN samples, and among the different types of berries blackcurrant presented the highest content of TPC, Flavo and ACY; the lowest values were registered for blueberry (TPC and ACY) and raspberry (Flavo). Our results are in agreement with those previously reported that highlighted a remarkable reduction of polyphenols in the IN fraction, compared to the OUT samples, in different berries, as for anthocyanins in strawberry and red wine [24,28], for total polyphenols and anthocyanins in wild blueberries and chockberry juice [25,26], for total polyphenols in cranberry bean extract and blackberry [27,30].

It is widely accepted that the antioxidant power of fruits strictly depends on the presence of efficient oxygen radical scavengers, such as vitamin C and phenolic compounds. Indeed, highly reactive species such as polyphenols act in plants as antioxidants and protective agents against several sources of damage (UV, pathogens, etc.). In the last years, the capacity of these compounds to scavenge free radicals and/or limit their formation has been accepted as the main mechanism through which they could counteract oxidative stress, preventing the onset and development of degenerative diseases [33,34]. However, polyphenols presented a very low bioavailability, with <1% of the ingested amount reaching the plasma: after consumption of polyphenol-rich foods or beverages the maximum concentrations of polyphenols found in the blood tend to be around 0.1–1 μM, much lower than the concentrations regularly used in vitro [35]. Therefore, phenolics may induce endogenous antioxidant defense systems through modulation of gene expression, counteracting oxidative stress indirectly. In addition, metabolites derived from colon microbiota activity have greater chemical stability than the parent compounds and might be present in the plasma at much higher concentrations and could, therefore play an important role in physiological effects. Despite this, the antioxidant capacity of fruit or individual components continues to represent a useful parameter to couple with other measurements. In the present work, the total antioxidant capacity of the different cultivars of each berry was evaluated after the in vitro gastrointestinal digestion process by the TEAC and FRAP assays (Table 2). Similar to the results obtained for the phytochemical composition, the OUT fractions presented higher values than the IN samples, and among berries, blackcurrant was the berry with higher TAC, measured by both TEAC and FRAP assays. On one side, these results are in accordance with those that reported a significant reduction of TAC after in vitro digestion [25,26,30], on the other side they are not surprising because of the strictly relationship between bioactive compound contents and antioxidant capacity of food. 

Cancer is one of the leading causes of death worldwide, and about 14.1 million of new cancer cases and 8.2 million of cancer deaths occurred in 2012 [36]. Many studies have shown that berry extracts, berry-derived products, or berry purified phenols exert significant in vitro and in vivo anti-tumor activity in different types of cancer, but only very few works focused on digested berry extracts [25,26,29]. For these reasons, we evaluated the cytotoxicity activities of digested fractions on HepG2 cells, assessing for each berry the cultivar with higher TPC and Flavo (“Loch Ness” for blackberry, “Ben Finlay” for blackcurrant, “Misty” for blueberry, “Glen Ericht” for raspberry, and “Romina” for strawberry). A dose- and time-dependent cytotoxic effect was found for all the tested berries, especially at 48 and 72 h of incubation. Results showed a dose- and time-dependent cytotoxic effect of the “IN” fractions after 24 h of incubation. These findings are in accordance with the few published data, which demonstrated a dose- and time-dependent cytotoxic effect of different berry digested extracts. For example, digested extracts from wild blueberry showed cell growth inhibition potential on human colorectal cancer cell line (HT 29), while digested raspberry extracts were effective in attenuating toxicity caused by ethyl carbamate in Caco-2 cells [25,29]. In the same way, chokeberry digested juice reduced proliferative rate by approximately 25% on Caco-2 cells, respect to the control, as well as digested Chinese bayberry exhibited dose-dependent relationship against HepG2 proliferation [26,37].

## 4. Materials and Methods

### 4.1. Standard and Reagents

2,2′-Azinobis(3-ethylbenzothiazolne-6-sulfonic acid) diammonium salt, ferrous sulphate and all other reagents and solvents were purchased from Sigma-Aldrich Chemie GmbH (Steinheim, Germany). 

### 4.2. Berry Sample

The study was performed on strawberry fruits of “Romina” and AN00.239.55 and AN07.216.60, a cultivar and 2 advanced selections of from the Ancona University breeding program [38], a clone of wild *F. vesca*—white fruit and of “Sabrina,” an international cultivar from a Spanish breeding program. The plants were grown in open field conditions using the standard cultivation conditions described in [38]. Ripe fruit samples were collected at the 2nd and 3rd harvest and frozen until the treatment for the in vitro digestion.

Samples of black currant cvs. Ben Finlay, Ben Maia, Ben Starav and Big Ben, all from the breeding program at the James Hutton Institute, Dundee, Scotland, were harvested at full ripeness from unirrigated open field plots grown under standard cultivation conditions. Samples were frozen immediately after harvest to await analysis. For Rubus berries, samples of the red raspberry cvs. Glen Ericht (bred in Scotland) and Tulameen (from Canada) were collected during the peak cropping season from breeding plots grown under polytunnels at the James Hutton Institute, using standard commercially based cultivation methods. Additionally, samples from the Scottish breeding lines 00123A7, 0304F6 and the yellow fruited 2J19 were also collected. All plots were grown under standard cultivation conditions. For the blackberry cv. Loch Ness from James Hutton and the Rubus species R. brigantinus and R. vagabundus, samples were collected from open field plots grown under standard cultivation conditions at full ripeness at National Institute for Agrarian and Veterinary Research Experimental Farm, Odemira. In Vaccinium, the cvs. Biloxi, Misty and Georgiagem were all Southern Highbush types were collected from open field plots grown under standard cultivation conditions at full ripeness at National Institute for Agrarian and Veterinary Research Experimental Farm, Odemira.

#### In Vitro Digestion

The in vitro digestion of berry samples was performed as previously described by McDougall et al. [24]. Briefly, the method consisted on reproducing gastrointestinal conditions in two different phases. First, samples were incubated with pepsin-HCl during 2 h for simulating gastric conditions (pH ~ 1.8) and then, with pancreatin and bile salts for another 2 h for simulating intestinal digestion (pH ~ 7.8); both phases at 37 °C in a shaking water bath. During the intestinal phase a dialysis membrane (molecular weight cut-off of 12,000 Da) was introduced in a beaker containing an aliquot of the post-gastric fraction. Subsequently, samples were concentrated under vacuum at 40 °C and stored at −80 °C until analysis.

### 4.3. Berry Phytochemical Characterization

For phytochemical characterization and TAC determination the extracts obtained after the in vitro digestion process were resuspended in Milli-Q water containing 0.1% methanol and subjected to an ultrasonic bath for 10 min at room temperature prior to analyses.

The TPC was measured by Folin-Ciocalteu method [39], TFC by the aluminum chloride spectrophotometric method [40], while total anthocyanin content of the digested samples was determined using a modified pH differential method previously described [41].

#### Total Antioxidant Capacity

The TAC of the berry digested samples was determined by two methods: the TEAC and the FRAP assays. 

The TEAC assay was carried out according to the modified method of Re and co-workers [42]. Briefly, the ABTS radical solution was prepared by reacting 7 mM ABTS aqueous stock solution with 2.45 mM K_2_S_2_O_8_ and maintained in the dark at 25 °C for 12 h before use. Previously to the analysis, the working solution was obtained by diluting the stock solution 1:50 with PBS buffer, pH 7.4. Then, 10 µL of alternatively blank, Trolox standard or MilliQ water diluted samples were added to 1 mL of the ABTS working solution into 1.5 mL eppendorfs. The mixture was vortexed for 20 s and after 1–3 min, the absorbance (A) was read at 734 nm, measuring the color inhibition of the ABTS radical.

Meanwhile, the FRAP assay was performed according to the protocol proposed by Deighton and co-workers (2000) [43]. Briefly, the FRAP reagent solution was prepared as follows: 10:1:1 of sodium acetate (300 mM, pH 3.6), TPTZ (10 mM in HCl 40 mM), and ferric chloride (20 mM). Briefly, 100 µL of sample or Trolox standard solution were added to 900 µL FRAP reagent. The mixture was vortexed, and, after 4 min, the absorbance was read at 593 nm against blank. 

Each sample was analyzed in three replicates and results are expressed as mM of Trolox equivalents (mM TEq). Data are reported as a mean value ± S.D. of four measurements.

### 4.4. Cell Culture

HepG2 cells were kindly provided by the Biological Research Laboratory of the Sevilla University (Spain), and were grown in Dulbecco’s modified Eagle’s medium, supplemented with 10% fetal bovine serum, 100 IU/mL penicillin and 100 ug/mL streptomycin at 37 °C in a humidified atmosphere with 5% CO_2_. The cells were incubated for 24, 48, and 72 h with different concentrations of berry digested extracts (0–5000 µg DW/mL). 

#### Cell Viability Assay

Cell viability was determined by the MTT assay, which is based on the reduction of tetrazolium salt, 3-(4,5-dimethylthiazol-2-yl)-2,5-diphenytetrazolium bromide, by intracellular dehydrogenases of viable living cells, leading to the formation of purple formazan crystals [44]. After incubation, fibroblasts were washed twice with PBS and incubated with a salt solution of MTT at a concentration of 0.5 mg/mL for 2 h at 37 °C. Then the medium was removed, and the crystals were dissolved in DMSO. The optical density of the suspension was read at 550 nm using a microplate reader (Synergy HT, Biotek, Winooski, VT, USA). Cell viability was expressed as a percentage of live cells compared to controls. The data reported represent average values from at least three independent experiments.

### 4.5. Statistical Analysis

Statistical analyses were performed using STATISTICA software (Statsoft Inc., Tulsa, OK, USA). Data were subjected to one-way analysis of variance for mean comparison, significant differences were calculated according to HSD Tukey’s multiple range test. Data were reported as mean ± SD. Differences at *p* < 0.05 were considered statistically significant.

## 5. Conclusions

Popularly consumed berries include blackberries, raspberries, blueberries, cranberries, raspberries, and strawberries. These soft fruits contain high levels of phenols that exert in vivo biological activities and can have complementary and overlapping mechanisms of action. In the last few years, several researchers have used in vitro digestion methods to analyze structural changes, and digestibility of foods, indicating that in vitro digestion systems are common and useful tools for food analyses. In the present work, we have compared the phytochemical composition and the total antioxidant capacity of different berries following the gastrointestinal digestion, and we have demonstrated that, although a large proportion of phenolics undergo transformation during digestion, berry polyphenols continue to exert antiproliferative effects. In particular, the most promising berry was black currant, which presented the higher content of polyphenols and total antioxidant capacity after in vitro digestion and consequently the highest cytotoxic effects on human hepatoma cells. However, the main limitation of the method here described is the complete lack of microbial transformation that occurs in the large intestine and is essential for polyphenols metabolism. In addition, further studies are needed to describe the impact of the digestion on the relative chemistries, to elucidate the mechanisms of transport and metabolism of these compounds, to evaluate the in vitro–in vivo correlations with well-defined systems, and to analyze the advantages and disadvantages of in vitro digestion models.

## Figures and Tables

**Table 1:** Phytochemical composition of the “IN” and “OUT” samples from different berries following the in vitro digestion.

Berry Variety	TPC (mg GAEq/g DW)		Flavo (mg CEq/g DW)		ACY (mg PgEq/g DW)	
*Blackberry*	*IN*	*OUT*	*IN*	*OUT*	*IN*	*OUT*
Loch Ness	0.46 ± 0.00 ^a^	1.28 ± 0.02 ^a^*	0.87 ± 0.04 ^a^	1.88 ± 0.00 ^a^*	0.97 ± 0.08 ^a^	1.19 ± 0.06 ^a^*
R. brigantinus	0.25 ± 0.00 ^b^	1.30 ± 0.05 ^a^*	0.43 ± 0.02 ^b^	1.63 ± 0.00 ^b^*	0.24 ± 0.03 ^b^	0.48 ± 0.04 ^b^*
R. vagabundus	0.22 ± 0.01 ^b^	0.93 ± 0.05 ^b^*	0.48 ± 0.02 ^b^	1.52 ± 0.01 ^c^*	0.27 ± 0.01 ^b^	0.35 ± 0.05 ^c^
*Mean*	0.32 ± 0.12	1.17 ± 0.19	0.59 ± 0.03	1.68 ± 0.00	0.50 ± 0.04	0.67 ± 0.05
*Blackcurrant*	*IN*	*OUT*	*IN*	*OUT*	*IN*	*OUT*
Ben Finlay	0.64 ± 0.02 ^a^	1.10 ± 0.05 ^a^	1.18 ± 0.02 ^a^	2.35 ± 0.15 ^a^*	1.29 ± 0.03 ^b^	1.81 ± 0.04 ^b^*
Ben Maia	0.24 ± 0.00 ^d^	0.38 ± 0.01 ^b^*	0.50 ± 0.00 ^c^	0.66 ± 0.00 ^c^*	0.89 ± 0.04 ^c^	0.98 ± 0.03 ^c^
Ben Starav	0.49 ± 0.00 ^b^	1.17 ± 0.03 ^a^*	1.01 ± 0.02 ^b^	2.32 ± 0.05 ^a^*	2.25 ± 0.09 ^a^	3.41 ± 0.01 ^a^*
Big Ben	0.29 ± 0.01 ^c^	0.41 ± 0.00 ^b^*	0.54 ± 0.01 ^c^	0.75 ± 0.01 ^b^*	0.71 ± 0.01 ^d^	0.88 ± 0.01 ^d^*
*Mean*	0.41 ± 0.17	0.77 ± 0.39	0.81 ± 0.02	1.52 ± 0.05	1.28 ± 0.04	1.77 ± 0.02
*Blueberry*	*IN*	*OUT*	*IN*	*OUT*	*IN*	*OUT*
Biloxi	0.05 ± 0.002 ^c^	0.25 ± 0.03 ^b^*	0.36 ± 0.02 ^c^	1.50 ± 0.24 ^b^*	0	0.07 ± 0.02 ^c^*
Georgiagem	0.11 ± 0.00 ^b^	0.35 ± 0.03 ^ab^*	0.52 ± 0.01 ^b^	1.50 ± 0.03 ^b^*	0.12 ± 0.01 ^a^	0.40 ± 0.11 ^a^*
Misty	0.14 ± 0.00 ^a^	0.44 ± 0.00 ^a^*	1.15 ± 0.02 ^a^	3.35 ± 0.02 ^a^*	0.03 ± 0.01 ^b^	0.17 ± 0.01 ^b^*
*Mean*	0.10 ± 0.04	0.35 ± 0.08	0.68 ± 0.01	2.12 ± 0.10	0.05 ± 0.01	0.21 ± 0.05
*Raspberry*	*IN*	*OUT*	*IN*	*OUT*	*IN*	*OUT*
Glen Ericht	0.31 ± 0.08 ^a^	0.50 ± 0.00 ^a^	0.53 ± 0.01 ^a^	0.91 ± 0.00 ^a^*	0.59 ± 0.00 ^b^	0.87 ± 0.01 ^b^*
Tulameen	0.26 ± 0.01 ^b^	0.35 ± 0.00 ^b^*	0.52 ± 0.02 ^a^	0.88 ± 0.18 ^a^*	1.02 ± 0.01 ^a^	1.21 ± 0.12 ^a^*
00123A7	0.17 ± 0.00 ^c^	0.31 ± 0.00 ^c^*	0.29 ± 0.01 ^b^	0.58 ± 0.01 ^b^*	0.33 ± 0.06 ^c^	0.60 ± 0.01 ^c^*
0304F6	0.16 ± 0.00 ^c^	0.32 ± 0.00 ^c^*	0.25 ± 0.00 ^c^	0.52 ± 0.00 ^c^*	0.15 ± 0.01 ^d^	0.37 ± 0.00 ^d^*
2J19 Yellow	0.16 ± 0.00 ^c^	0.26 ± 0.00 ^d^*	0.30 ± 0.02 ^b^	0.48 ± 0.03 ^c^*	0	0
*Mean*	0.22 ± 0.06	0.35 ± 0.08	0.38 ± 0.01	0.67 ± 0.05	0.42 ± 0.01	0.61 ± 0.03
*Strawberry*	*IN*	*OUT*	*IN*	*OUT*	*IN*	*OUT*
*F. vesca*—white	0.29 ± 0.01 ^a^	0.78 ± 0.04 ^a^*	0.39 ± 0.02 ^b^	1.40 ± 0.01 ^a^*	0	0
Romina	0.29 ± 0.03 ^a^	0.71 ± 0.09 ^a^*	0.36 ± 0.00 ^c^	1.02 ± 0.02 ^c^*	0.37 ± 0.01 ^a^	0.67 ± 0.01 ^a^*
Sabrina	0.21 ± 0.00 ^b^	0.59 ± 0.01 ^ab^*	0.42 ± 0.00 ^b^	0.77 ± 0.02 ^e^*	0.09 ± 0.05 ^b^	0.33 ± 0.02 ^c^*
AN 00.239.55	0.23 ± 0.00 ^ab^	0.51 ± 0.01 ^b^*	0.45 ± 0.00 ^a^	1.18 ± 0.00 ^b^*	0.34 ± 0.04 ^a^	0.52 ± 0.01 ^b^*
AN 07.216.60	0.24 ± 0.00 ^ab^	0.41 ± 0.02 ^b^*	0.34 ± 0.00 ^c^	0.84 ± 0.02 ^d^*	0.12 ± 0.00 ^b^	0.13 ± 0.01 ^d^
*Mean*	0.26 ± 0.04	0.60 ± 0.15	0.39 ± 0.01	1.04 ± 0.02	0.18 ± 0.02	0.33 ± 0.01

Data were reported as mean ± SD. Significant differences (*p* < 0.05) between the different berry species are indicated with differing lower-case letters in superscript (in a column). * Significant differences (*p* < 0.05) between IN and OUT fractions for each berry for the same parameter analyzed. Abbreviations: TPC, Total Phenolic Content; Flavo, Flavonoids; ACY, anthocyanins; DW, dried weight; GAEq, gallic acid equivalents; CEq, catechin equivalents; PgEq, pelargonidin-3-glucoside equivalemts.

**Table 2:** Total antioxidant capacity of the “IN” and “OUT” samples from different berries following the in vitro digestion.

Berry Variety	TEAC (µmol Teq/g DW)		FRAP (µmol Teq/g DW)	
*Blackberry*	*IN*	*OUT*	*IN*	*OUT*
Loch Ness	9.54 ± 1.53 ^a^	22.19 ± 0.72 ^b^*	10.00 ± 0.23 ^a^	14.70 ± 1.39 ^b^*
R. brigantinus	7.31 ± 0.36 ^b^	28.75 ± 1.55 ^a^*	8.24 ± 0.08 ^b^	22.24 ± 0.59 ^a^*
R. vagabundus	8.48 ± 1.51 ^ab^	32.93 ± 4.81 ^a^*	10.31 ± 0.19 ^a^	22.41 ± 1.73 ^a^*
*Mean*	8.44 ± 1.13	27.96 ± 2.36	9.51 ± 0.17	19.79 ± 1.23
*Blackcurrant*	*IN*	*OUT*	*IN*	*OUT*
Ben Finlay	41.49 ± 1.30 ^a^	99.04 ± 1.17 ^a^*	15.90 ± 0.36 ^b^	32.09 ± 2.01 ^a^*
Ben Maia	21.95 ± 2.35 ^b^	23.63 ± 0.74 ^d^	9.31 ± 0.12 ^c^	15.70 ± 0.68 ^b^*
Ben Starav	44.20 ± 2.65 ^a^	91.05 ± 0.29 ^b^*	16.62 ± 0.07 ^a^	33.16 ± 0.39 ^a^*
Big Ben	18.70 ± 0.20 ^b^	29.33 ± 1.08 ^c^*	9.44 ± 0.34 ^c^	15.59 ± 1.82 ^b^*
*Mean*	31.59 ± 1.62	60.76 ± 0.82	12.82 ± 0.22	24.13 ± 1.22
*Blueberry*	*IN*	*OUT*	*IN*	*OUT*
Biloxi	3.46 ± 0.04 ^b^	7.76 ± 0.62 ^b^*	3.77 ± 0.01 ^c^	10.54 ± 0.04 ^c^*
Georgiagem	5.39 ± 0.12 ^a^	12.23 ± 0.64 ^a^*	6.41 ± 0.04 ^b^	14.87 ± 0.23 ^b^*
Misty	5.60 ± 0.19 ^a^	12.65 ± 0.03 ^a^*	7.24 ± 0.02 ^a^	20.33 ± 0.10 ^a^*
*Mean*	4.82 ± 0.11	10.88 ± 0.43	5.81 ± 0.02	15.25 ± 0.12
*Raspberry*	*IN*	*OUT*	*IN*	*OUT*
Glen Ericht	20.38 ± 0.09 ^a^	34.57 ± 2.21 ^a^*	8.07 ± 0.03 ^b^	13.61 ± 0.16 ^a^*
Tulameen	20.00 ± 0.24 ^a^	29.51 ± 0.32 ^b^*	8.32 ± 0.08 ^a^*	2.39 ± 0.52 ^d^
00123A7	15.15 ± 0.92 ^b^	20.30 ± 0.62 ^c^*	4.61 ± 0.32 ^c^	10.57 ± 1.94 ^b^*
0304F6	12.64 ± 0.20 ^c^	21.15 ± 0.20 ^c^*	4.32 ± 0.04 ^c^	7.79 ± 0.20 ^c^*
2J19 Yellow	15.99 ± 0.00 ^b^	17.47 ± 0.23 ^d^*	4.04 ± 0.24 ^c^	7.47 ± 1.40 ^c^*
*Mean*	16.83 ± 0.29	24.60 ± 0.72	5.87 ± 0.14	8.37 ± 0.84
*Strawberry*	*IN*	*OUT*	*IN*	*OUT*
*F. vesca*—white	10.53 ± 0.5 ^a^	25.12 ± 0.19 ^a^*	15.97 ± 1.24 ^a^	42.69 ± 7.39 ^a^*
Romina	6.36 ± 0.48 ^cd^	20.33 ± 0.43 ^c^*	9.46 ± 0.08 ^d^	21.45 ± 0.42 ^b^*
Sabrina	5.64 ± 0.23 ^d^	22.48 ± 0.14 ^b^*	7.64 ± 0.13 ^e^	15.55 ± 0.83 ^d^*
AN 00.239.55	7.77 ± 0.07 ^b^	21.80 ± 0.18 ^b^*	12.79 ± 0.97 ^b^	25.50 ± 5.12 ^b^*
AN 07.216.60	7.89 ± 0.53 ^b^	22.00 ± 1.44 ^b^*	10.89 ± 0.13 ^c^	19.15 ± 0.75 ^c^*
*Mean*	7.64 ± 0.36	22.35 ± 0.48	11.35 ± 0.51	24.87 ± 2.90

Data were reported as mean ± SD. Significant differences (*p* ˂ 0.05) between the different varieties of each berry are indicated with differing lower-case letters in superscript (in a column). * Significant differences (*p* ˂ 0.05) between IN and OUT fractions for each variety for the same parameter analyzed. Abbreviations: TEAC, Trolox Equivalent Antioxidant Capacity; FRAP, Ferric Reducing Antioxidant Power; µmol TEq, µmolesTrolox Equivalents; DW, digested weight.

## Abbreviations

ACY	Anthocyanins
DW	digested weight
Flavo	flavonoid content
FRAP	Ferric Reducing Antioxidant Power
GI	gastrointestinal
HepG2	human hepatocellular carcinoma cells.
HT 29	Human colorectal cancer cell line
MTT	3-(4,5-dimethylthiazol-2-yl)-2,5-diphenyltetrazolium bromide
TAC	Total antioxidant capacity
TEAC	Trolox Equivalent Antioxidant Capacity
TPC	Total phenolic content
